# A phase 3 randomized, placebo-controlled study assessing the efficacy and safety of epoetin-α in anemic patients with low-risk MDS

**DOI:** 10.1038/s41375-018-0118-9

**Published:** 2018-03-30

**Authors:** Pierre Fenaux, Valeria Santini, Maria Antonietta Aloe Spiriti, Aristoteles Giagounidis, Rudolf Schlag, Atanas Radinoff, Liana Gercheva-Kyuchukova, Achilles Anagnostopoulos, Esther Natalie Oliva, Argiris Symeonidis, Mathilde Hunault Berger, Katharina S. Götze, Anna Potamianou, Hari Haralampiev, Robert Wapenaar, Iordanis Milionis, Uwe Platzbecker

**Affiliations:** 1 Hôpital St. Louis, Assistance Publique Hôpitaux de Paris, Université Paris Diderot, Paris, France; 2 Hematology AOU Careggi, University of Florence, Florence, Italy; 3 Sapienza Università di Roma, Rome, Italy; 4 Marien Hospital, Düsseldorf, Germany; 5 Praxisklinik Würzburg, Würzburg, Germany; 6 University Hospital Sveti Ivan Rislki, Sofia, Bulgaria; 7 Varna Clinic of Haematology, Varna, Bulgaria; 8 George Papanicolaou Hospital, Thessaloniki, Greece; 9 Grande Ospedale Metropolitano Bianchi-Melacrino-Morelli, Calabria, Italy; 10 Department of Hematology, Medical School, University of Patras, Patras, Greece; 11 CHU Angers, Angers, France; 12 Technische Universität München, Munich, Germany; 13 EMEA Medical Affairs, Janssen-Cilag, Neuss, Germany; 14 EMEA Medical Affairs, Covance CoSource on assignment with Janssen, Sofia, Bulgaria; 15 Biostatistics, Janssen-Cilag BV, Breda, The Netherlands; 16 EMEA Medical Affairs, Janssen-Cilag Pharmaceutical SACI, Athens, Greece; 17 Medizinische Klinik und Poliklinik I, Dresden, Germany

## Abstract

Erythropoiesis-stimulating agents are first choice for treating anemia in low-risk MDS. This double-blind, placebo-controlled study assessed the efficacy and safety of epoetin-α in IPSS low- or intermediate-1 risk (i.e., low-risk) MDS patients with Hb ≤ 10.0 g/dL, with no or moderate RBC transfusion dependence (≤4 RBC units/8 weeks). Patients were randomized, 2:1, to receive epoetin-α 450 IU/kg/week or placebo for 24 weeks, followed by treatment extension in responders. The primary endpoint was erythroid response (ER) through Week 24. Dose adjustments were driven by weekly Hb-levels and included increases, and dose reductions/discontinuation if Hb > 12 g/dL. An independent Response Review Committee (RRC) blindly reviewed all responses, applying IWG-2006 criteria but also considering dose adjustments, drug interruptions and longer periods of observation.

A total of 130 patients were randomized (85 to epoetin-α and 45 to placebo). The ER by IWG-2006 criteria was 31.8% for epoetin-α vs 4.4% for placebo (*p* < 0.001); after RRC review, the ER was 45.9 vs 4.4% (*p* < 0.001), respectively. Epoetin-α reduced RBC transfusions and increased the time-to-first-transfusion compared with placebo.

Thus, epoetin-α significantly improved anemia outcomes in low-risk MDS. IWG-2006 criteria for ER may require amendments to better apply to clinical studies.

## Key points (short bulleted statements of relevant outcomes)

- Epoetin-α improved erythroid response, reduced the percentage of patients requiring red blood cell transfusion and increased the time-to-first-transfusion compared with placebo
- Epoetin-α was well tolerated in anemic patients with low- and intermediate-1 risk MDS

## Introduction

MDS are clonal myeloid disorders, characterized by ineffective hematopoiesis, leading to peripheral blood cytopenias and an increased risk of progression to acute myeloid leukemia (AML) [1]. Myelodysplastic syndromes (MDS) are classified prognostically by the International Prognostic Scoring System (IPSS). In low-risk MDS (i.e., IPSS low- or intermediate-1-risk), anemia is the predominant feature, leading to red blood cell (RBC) transfusion requirement, poor quality of life (QoL), and worsening of comorbidities [2].

Until recently, lenalidomide was the only drug approved in the EU to treat anemia associated with low-risk MDS, but only in patients with 5q deletion; however, many studies have evaluated erythropoiesis-stimulating agents (ESAs) in all subgroups of patients with MDS [3–5]. Recombinant human erythropoietin alfa (epoetin-α; Eprex^®^) stimulates proliferation of RBC precursors and inhibits their apoptosis in MDS [6]. In the EU, epoetin-α is indicated to treat symptomatic anemia associated with chronic renal failure, chemotherapy-treated adult cancer patients at risk of transfusion, adults in a pre-donation program to increase the yield of autologous blood, and patients prior to orthopedic surgery at risk of transfusion complications [7].

Epoetin-α has been used to treat anemia in low-risk MDS patients, primarily in non-randomized, single-arm studies [8–11]. A well-designed, randomized, double-blind study with sufficient size and duration was required to compare epoetin-α with placebo in improving anemia outcomes in patients with low-risk MDS.

We designed a phase 3 multicenter double-blind placebo-controlled study to evaluate epoetin-α as treatment for anemia in low-risk MDS. Evaluation of our results led us to reconsider some of the International Working group (IWG)-2006 criteria for erythroid response (ER).

## Methods

### Study design

Adults with MDS were randomized in a 2:1 ratio to receive either epoetin-α or placebo, at 29 sites in six countries across Europe. Inclusion criteria were de novo MDS according to WHO classification, and IPSS low- or intermediate-1 (low-risk) at screening, hemoglobin (Hb) ≤10 g/dL (10.5 g/dL in case of preceding transfusion), serum erythropoietin <500 mU/mL, transfusion requirement ≤4 RBC units/8 weeks, ECOG performance status ≤2, and adequate iron, B12 and folate levels. Exclusion criteria were therapy-related MDS, uncontrolled hypertension, prior treatment with any ESA or interventional agents, and a history of pure red cell aplasia. Randomization was stratified according to transfusion requirement (yes vs no) and serum erythropoietin levels (≥200 mU/mL vs <200 mU/mL).

Epoetin-α was administered weekly, subcutaneously at an initial dose of 450 IU/kg (up to 40,000 IU total dose) or matching volume of placebo. Hb was measured weekly, either centrally or at local sites. At week 8 the dose could be increased up to 1050 IU/kg (up to 80,000 IU total dose) or matching volume of placebo in patients not achieving an ER. If Hb levels increased to >12 g/dL, or there was a rapid increase in Hb levels (>2 g/dL over any 4-week period), treatment with epoetin-α was interrupted. Stepwise dose adjustments were permitted for patients not achieving ER, and dose reduction/interruption driven by weekly Hb regardless of ER status (Table S1). At the end of Week 24, responders could enter the double-blind extension phase through Week 48 continuing with the same dosing rules (Figure S1). Patients completing or discontinuing treatment were followed for a further 4 weeks.

### Endpoints

The primary endpoint was ER through Week 24, according to IWG-2006 criteria [12]. Following difficulties in assessing response using the IWG-2006 criteria—due in particular to the per label dose adjustment of epoetin-α—an independent response review committee (RRC) reviewed blinded data on dosing, transfusions, Hb levels and somewhat modified IWG-2006 criteria for ER as follows: (i) patients with an increase in Hb level by at least 1.5 g/dL lasting less than 8 weeks due to epoetin-α discontinuation were considered responders if, when restarting epoetin-α at lower dose, Hb still increased by at least 1.5 g/dL, (ii) in transfused patients, the baseline Hb value was taken before the last transfusion preceding enrollment (rather than after), and (iii) if discrepancies were observed between local and centralized Hb levels, the latter should be used to evaluate response and its duration (Table S2).

Secondary endpoints included duration of ER through 48 weeks, time-to-RBC-transfusion, the number of RBC units transfused, and QoL. QoL questionnaires included the Functional Assessment of Cancer Therapy-Anemia/Fatigue (FACT-An), EuroQol 5-dimension (EQ-5D-3L). AEs were collected for patients who received ≥1 dose of study medication. An independent data and safety monitoring committee reviewed unblinded safety and efficacy data.

### Open-label access study

Patients in Bulgaria, Greece, and Germany responding to treatment with epoetin-α at Week 48, and non-responders by Week 24 treated with placebo were eligible for an open-label study, where they could receive, or continue to receive, epoetin-α at the end of their participation in the EPOANE3021 study. The duration was 6 months in Germany and Greece, and up to 1 year after the last patient had enrolled in Bulgaria, or until early discontinuation). Patients were given an initial dose of epoetin-α 450 IU/kg and a maximum dose of 40,000 IU/kg weekly for the first 8 weeks, after which the protocol-defined dosing schedule and modifications were applied, or continued the same dose when entering the open-label study. Patients were withdrawn from the study when treated with the maximum dose for 8 weeks without response.

### Statistical analysis

Sample size was based on an expected response rate of 35% and 10% in the epoetin-α and placebo groups, respectively. Using a Fisher exact test with a 0.05 two-sided significance level, corrected for a 10% drop out rate, ≥125 patients (83 epoetin-α, 42 placebo) were required to achieve ≥80% power. All statistical tests were two-sided at a significance level of 0.05. Modified intent to treat (mITT—patients with ≥1 dose and one post-baseline assessment) and per protocol (PP—no major protocol deviations during the first 24 weeks) analyses were used for all efficacy analyses. For ER, Fisher’s, and Cochran–Mantel–Haenszel test were used (the latter taking into account stratification factors and IPSS risk category at screening). Between-group comparisons were tested using the Fisher exact test or Wilcoxon two-sample test.

## Results

### Patient characteristics

Of 130 patients randomized between September 2011 and January 2014, 85 were assigned to epoetin-α and 45 to placebo. Baseline patient clinical characteristics were well balanced. The median age was 75 years, and 54.6% of patients were men (Table 1 and Table S3). Overall, 50% of patients had an ECOG score of 1.

**Table 1:** Baseline patient characteristics

Baseline characteristics	Placebo *n* = 45	Epoetin-α *n* = 85	Total *N* = 130
Age (years), median	75	85	75
Range (years)	36–87	40–94	36–94
Sex			
Male	25 (55.6%)	46 (54.1%)	71 (54.6%)
Female	20 (44.4%)	39 (45.9%)	59 (45.4%)
Body mass index (kg/m^2^)	*n* = 45	*n* = 84	*N* = 129
Mean (SD)	25.94 (4.486)	27.58 (4.550)	27.01 (4.578)
Median (range)	25.97 (16.1–36.3)	27.13 (18.2–40.5)	26.67 (16.1–40.5)
Hemoglobin (g/dL)			
Mean (SD)	9 (0.848)	9 (0.939)	
Median (range)	9 (6.9–10.5)	9 (6.8–11.0)	
Transfusions in 8 weeks prior to baseline visit			
Patients with transfusions (%)	22 (48.9%)	44 (51.8%)	
No. of transfusion events prior to baseline	36	75	
Total RBC units prior to visit	53	114	
RBC units required per patient receiving transfusions	2.4	2.6	
MDS subtype according to WHO classification	*n* = 44	*n* = 82	*N* = 126
RA	11 (24.4%)	7 (8.2%)	18 (13.8%)
RARS	2 (4.4%)	9 (10.6%)	11 (8.5%)
RCMD	21 (46.7%)	36 (42.4%)	57 (43.8%)
RCMD-RS	5 (11.1%)	12 (14.1%)	17 (13.1%)
RAEB-1	1 (2.2%)	10 (11.8%)	11 (8.5%)
RAEB-2	0	1 (1.2%)	1 (0.8%)
MDS-U	0	1 (1.2%)	1 (0.8%)
5q-	3 (6.7%)	2 (2.4%)	5 (3.8%)
AML	0	0	0
Not available	1 (2.2%)	4 (4.7%)	5 (3.8%)
MDS subtype according to FAB classification^a^	*n* = 44	*n* = 82	*N* = 126
RA	35 (77.8%)	46 (54.1%)	81 (62.3%)
RARS	7 (15.6%)	21 (24.7%)	28 (21.5%)
RAEB	1 (2.2%)	11 (12.9%)	12 (9.2%)
RAEB-t	0	0	0
CMML	1 (2.2%)	4 (4.7%)	5 (3.8%)
AML	0	0	0
Not available	0	0	0
IPSS risk category^b^	*n* = 45	*n* = 85	*N* = 130
Low	23 (51.1%)	35 (41.2%)	58 (44.6%)
Intermediate-1 (0.5–1.0)	22 (48.9%)	49 (57.6%)	71 (54.6%)
Intermediate-2 (1.5–2.0)	0	0	0
High (≥2.5)	0	0	0
Missing	0	1 (1.2%)	1 (0.8%)
*P* value		0.355	
ECOG score	*n* = 45	*n* = 85	*N* = 130
0—fully active	20 (44.4%)	35(41.2%)	55 (42.3%)
1—restricted but ambulatory	23 (51.1%)	42 (49.4%)	65 (50.0%)
2—ambulatory	2 (4.4%)	8 (9.4%)	10 (7.7%)
3—capable but confined to bed/chair	0	0	0
4—completely disabled	0	0	0

*5q-*   myelodysplastic syndromes associated with isolated del(5q), *AML*   acute myeloid leukemia, *CMML* chronic myelomonocytic leukemia, *FAB*   French-American-British, *IPSS*   International Prognostic Scoring System, *ECOG*   Eastern Cooperative Oncology Group, *MDS *myelodysplastic syndromes, *MDS-U*   myelodysplastic syndrome, unclassified, *RA*   refractory anemia, *RARS*   refractory anemia with ringed sideroblasts, *RAEB* refractory anemia with excess blasts, *RAEB-t* refractory anemia with excess blasts in transformation, *RBC* red blood cells, *RCMD *  refractory cytopenia with multilineage dysplasia, *RCMD-RS*   refractory cytopenia with multilineage dysplasia with ringed sideroblasts, *SD*   standard deviation, *WHO* World Health Organization

^a^According to FAB, CMML patients were marked as MDS subtype not available in the WHO classification

^b^One patient was missing the IPSS category at screening. The *p*value for treatment group differences are based on the Fisher exact test, two-sided

**Fig. 1:**
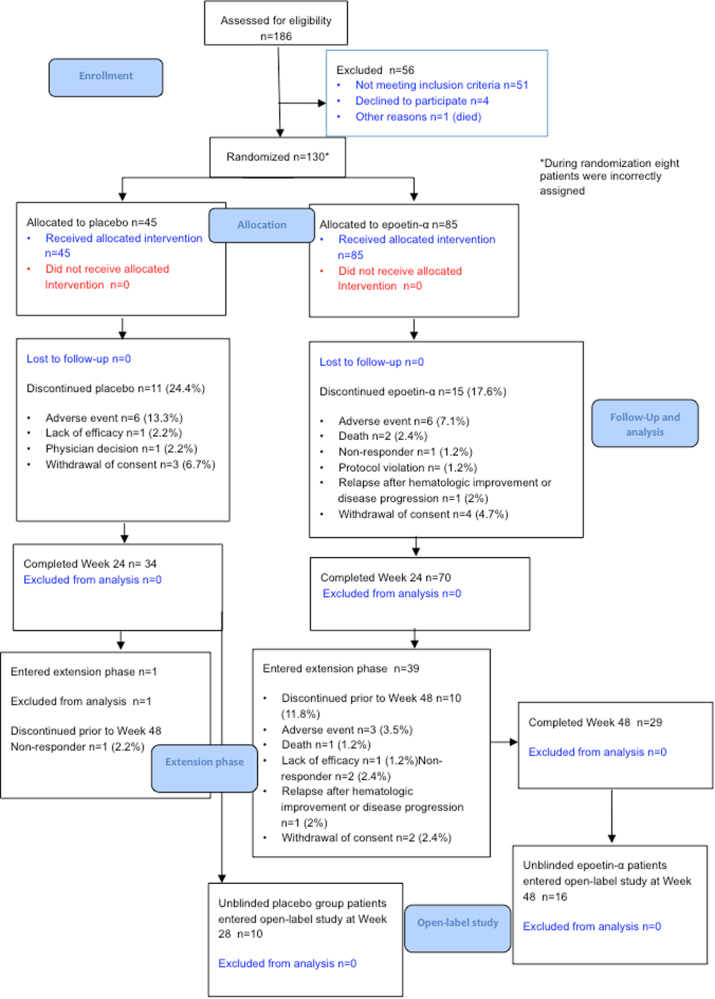
CONSORT flow diagram

After 24 weeks, 24.4% in the placebo group and 17.6% in the epoetin-α group had discontinued treatment (Fig. 1). The most common reasons for discontinuation were AEs (in 7.1% of the patients in the epoetin-α group vs 13.3% of those in the placebo group) and consent withdrawal (in 4.7 vs 6.7%) (Table S4).

Thirty-nine patients in the epoetin-α group and one patient in the placebo group entered the 24-week extension phase. The most common reasons for treatment discontinuation at any time during the 24-week extension phase were no response to treatment and AEs (Table S4).

The median weekly dose delivered was 730.4 IU/kg (range: 343–946) in the epoetin-α vs 850.0 IU/kg (range: 404–910) in the placebo groups. The epoetin-α dose was decreased in 54.1% receiving the drug versus 20.0% in those receiving placebo. The mean treatment duration for the epoetin-α group was 30.9 (standard deviation (SD) 14.04) weeks and 21.3 (SD 6.38) weeks for the placebo group (mITT).

### Efficacy results

#### ER based on strict IWG 2006 response criteria

The ER was 31.8% for the epoetin-α group vs 4.4% for the placebo group, (*P*<0.001; mITT) Table 2). All responders had a serum erythropoietin level of <200 mU/mL at baseline. In patients with no transfusion need, ER was 50% for epoetin-α vs 4.8% for placebo. In IPSS low-risk patients, ER was 45.7% in the epoetin-α vs 8.7% in the placebo group; in IPSS intermediate-1 20.4 vs 0%, respectively.

**Table 2:** Erythroid response at any time during the first 24 weeks (mITT and PP analyses)

ER	miTT analysis		PP analysis	
	Placebo *n* = 45	Epoetin-α *n* = 85	Placebo *n* = 21	Epoetin-α *n* = 32
Patients with erythroid response^a^ at any time during the first 24 weeks of the study	2 (4.4%)	27 (31.8%)	0	11 (34.4%)
*P* value^b^		<0.001		0.002
Patients with erythroid response by stratification group				
No transfusion and serum erythropoietin level less than 200 mU/mL^c^	1 (4.8%)	20 (50.0%)	0	8 (66.7%)
Transfusion and serum erythropoietin level less than 200 mU/mL^c^	1 (5.6%)	7 (22.6%)	0	3 (25.0%)
No transfusion and serum erythropoietin level at least 200 mU/mL	0	0	0	0
Transfusion and serum erythropoietin level at least 200 mU/mL	0	0	0	0
*P* value^d^		<0.001		0.001
Patients with erythroid response by IPSS risk category				
Low = 0^e^	2 (8.7%)	16 (45.7%)	0	7 (58.3%)
Intermediate-1 = 0.5–1.0^e^	0	10 (20.4%)	0	4 (20.0%)
Intermediate-2 = 1.5–2.0	0	0	0	0
High = ≥ 2.5	0	0	0	0
No IPSS at screening	0	1	0	0
*P* value^d^		<0.001		0.001
Percentage of patients with erythroid response at any time during the first 24 weeks of study for evaluable patients^f^	2 (4.4%)	27 (32.9%)	0	11 (34.3%)
	*High*		*Intermediate*	*Low*
ER according to Nordic Score Classification				
Responders	21 (44.7%)		6 (16.7%)	
Nonresponders	26 (55.3%)		30 (83.3%)	1 (100%)
*MDS subtypes*	*RA/RCMD*		*RARS/RCMD-RS*	
ER according to RA/RCMD and RARS/RCMD-RS MDS subtypes				
Responders	13 (30.2%)		8 (38.1%)	
Nonresponders	30 (69.8%)		13 (61.9%)	

*CMH*   Cochran–Mantel–Haenszel,* IPSS* International Prognostic Scoring Systems, *IWG*   International Working Group, *NR*  not reported; *RBC*   red blood cell, *RRC*   Response Review Committee

^a^Erythroid response assessed according to the IWG 2006 criteria: Hemoglobin increase by ≥1.5 g/dL or relevant reduction of RBC units transfused by an absolute number of at least four units every 8 weeks compared with the pretreatment transfusion number in the previous 8 weeks; responses must last at least 8 weeks

^b^*P* value for treatment group differences are based on the Fisher exact test, two-sided

^c^mITT analysis: The CMH *p*-value and percentages are based on the number of patients in that strata: placebo, Strata 1 = 20 and Strata 2 = 19; epoetin alfa, Strata 1 = 38 and Strata 2 = 33; PP analysis: placebo, Strata 1 = 8 and Strata 2 = 10; epoetin alfa, Strata 1 = 12 and Strata 2 = 12

^d^*P* value for treatment group differences are based on the CMH test, two-sided

^e^The CMH *P* value and percentages are based on the number of patients in that IPSS category: placebo, low 0 = 23 and intermediate-1 = 22; epoetin-α, low 0 = 35 and intermediate-1 = 49

^f^The denominator excludes patients who were determined by the RRC as not evaluable

#### ER based on the modified-IWG-2006 response criteria

Difficulties in interpretation of IWG-2006 criteria occurred (Table S5). First, patients who transiently discontinued epoetin-α due to Hb>12 g/dL or received a dose reduction following an increase of >2 g/dL in Hb over any 4-week period often did not meet IWG-2006 criteria, as their response was <8 weeks (due to drug discontinuation/dose reduction). Also, because transfused patients had, as per inclusion criteria, a RBC rate below 4 units/8 weeks, IWG-2006 criteria required both RBC-transfusion independence and an increase in Hb level by ≥1.5 g/dL. In addition, baseline Hb level is defined in IWG-2006 criteria as “average of ≥2 measurements (not influenced by transfusions), 1 week apart”. However, in a patient with low-transfusion requirement (e.g., every 6 weeks), baseline Hb levels are not the same in the 2 weeks before and the 2 weeks after transfusions. Also, some epoetin-α dose adjustments were based on local Hb levels (frequently measured with a portable photometer), which sometimes differed from Hb levels measured at the centralized laboratory. Finally, the baseline RBC-transfusion rate evaluated during the prior 8 weeks often did not reflect the actual long-term RBC-transfusion rate, which was more accurately captured by analyzing the prior 16 weeks rate.

Taking into account these considerations, the RRC blindly reviewed all cases, based on modified IWG 2006 response assessment. Using these criteria, the proportion of ER in the first 24 weeks was 45.9% in the epoetin-α treatment group vs 4.4% in the placebo group (*P* < 0.001).

### Other outcomes

Of the 27 responders, five patients discontinued treatment before week 48 while still in response (median duration 25 weeks, range 13–38). Nine patients were still responding at study end with a median duration of 40 weeks (range 24–46). The remaining 13 patients had relapsed while still on treatment, after a median of 19 weeks (range 8–44). Thus, the mean response duration in responders was 27.5 weeks, while the estimated median Kaplan–Meier estimated duration of response was 44 weeks.

**Fig. 2:**
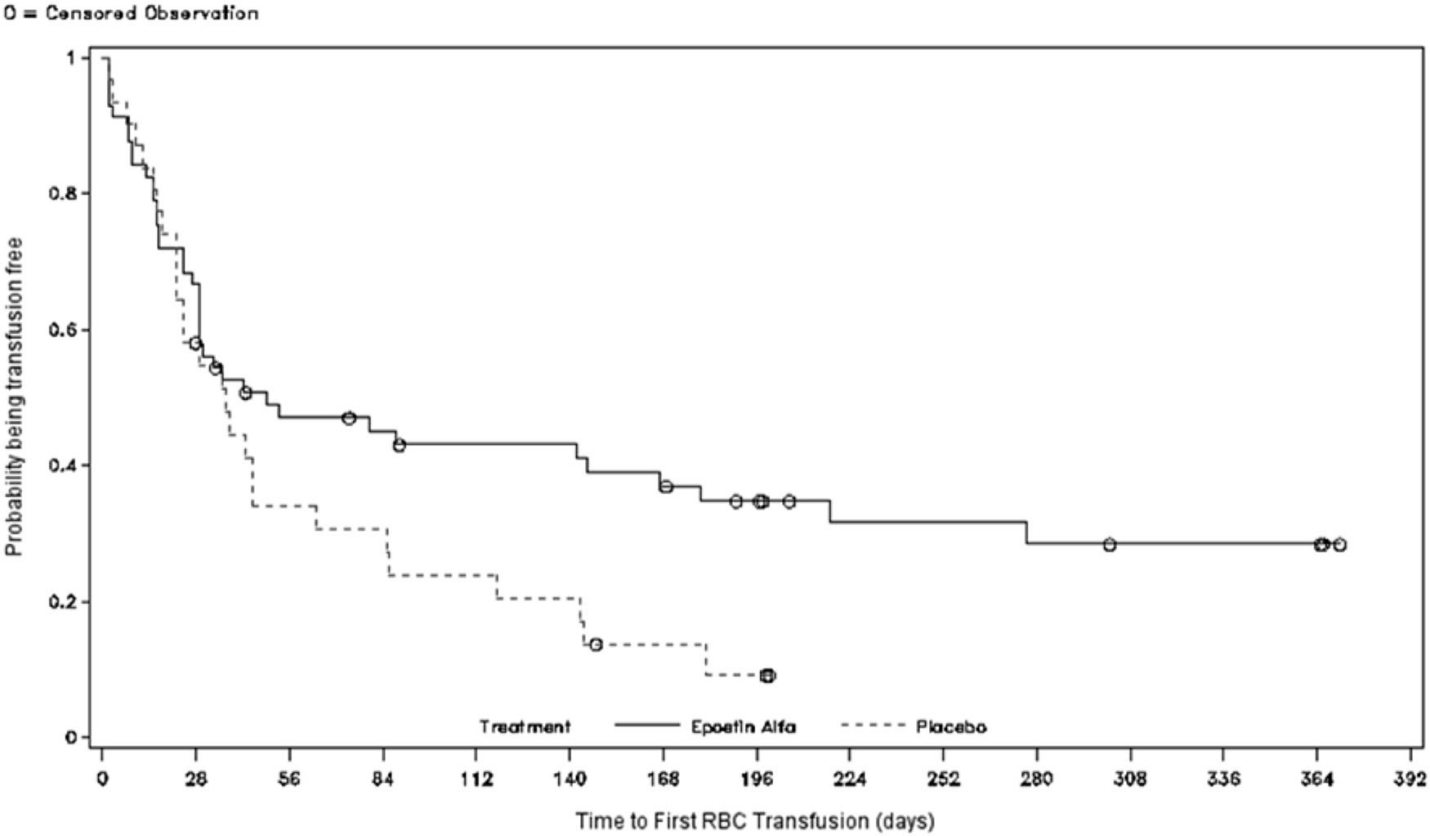
Time-to-first-red blood cell (RBC)-transfusions (mITT)

**Fig. 3:**
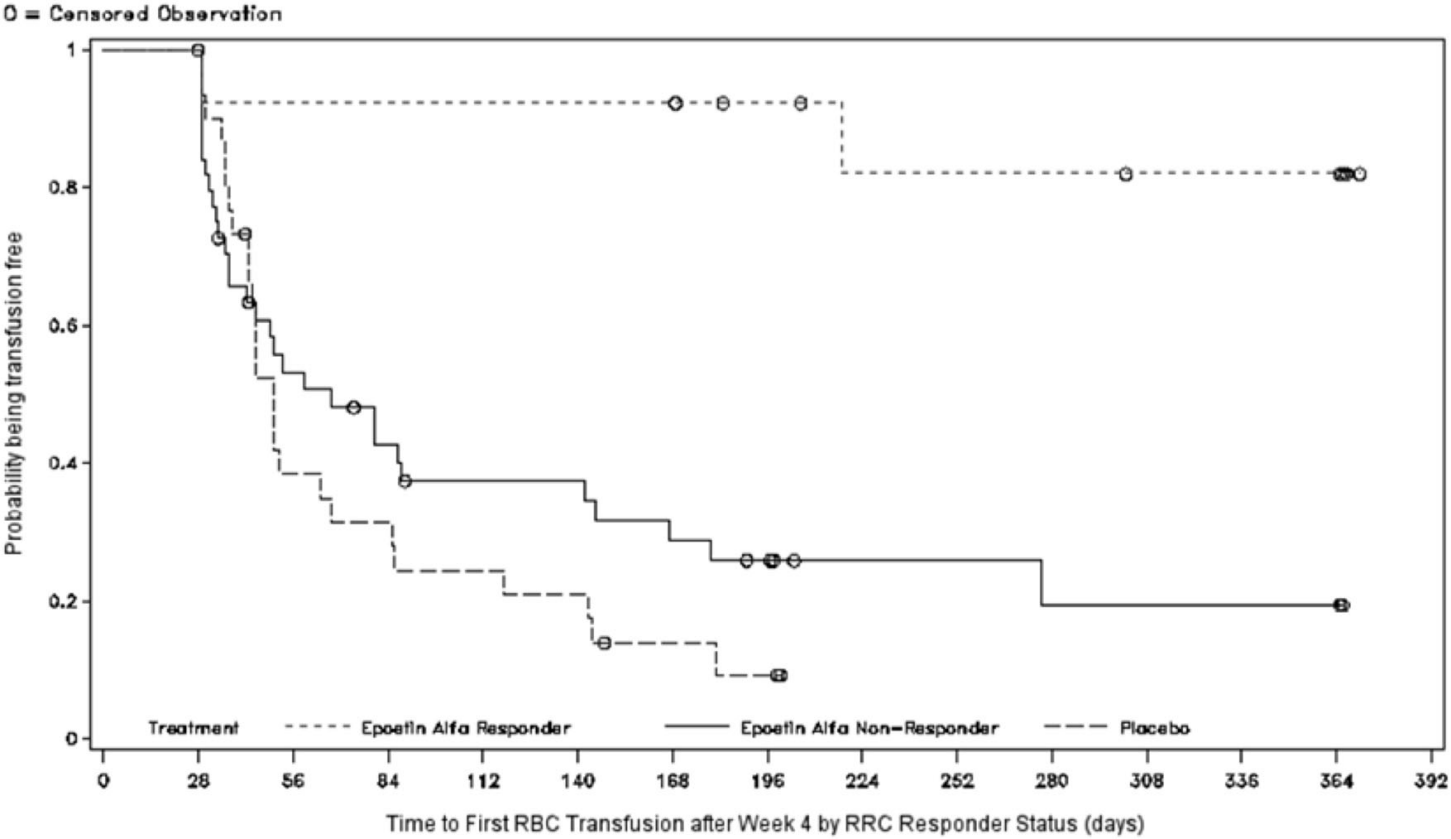
Time-to-first-red blood cell (RBC)-transfusions after week 4 by RRC responder status (mITT)

Figure 2 shows the time-to-first-RBC-transfusion between the treatment groups (Kaplan–Meier; mITT). There was a significant difference in time-to-first-transfusion in the epoetin-α group vs placebo group (log-rank test: median 7.0 vs 5.3 weeks, respectively *P* = 0.046; hazard ratio [HR] 1.653 (95% confidence interval [CI] 0.999–2.736). The separation between the two treatment groups began around Week 4: the difference in RBC transfusions occurring after Week 4 was a median 20.3 weeks (95% CI, 7.7–39.6) in the epoetin-α, and 7.1 weeks (95% CI 6.1–9.6) in the placebo groups (*P* = 0.007) (Fig. 3).

Between baseline and Week 24, 36 (42.4%) patients in the epoetin-α group received 163 transfusions (total RBC units = 266; units per patients = 7.4); and 26 (57.8%) patients in the placebo group received 125 transfusions (total RBC units = 196; units per subject = 7.5).

At Week 24, the mean increase from baseline in the Hb levels in the epoetin-α group was 1.04 g/dL and the mean decrease in the Hb levels in the placebo group was 0.07 g/dL (Table S7).

### QoL

There were no significant differences in QoL between the epoetin-α group and the placebo at any time point. QoL at Week 24 was significantly different between the responders in the epoetin-α group and the placebo group (EQ-5D index score *P* = 0.034).

### Safety results

During the 24 weeks of the randomized study (Tables 3 and 4), more patients in the placebo group (88.9%) reported one or more TEAE compared with the epoetin-α group (77.6%). At least one TEAE leading to permanent discontinuation of study treatment was reported in 10.6% of patients in the epoetin-α group and 13.3% of patients in the placebo group (Table 5). Similar numbers of patients reported at least one TEAE of toxicity grade 3 or grade 4 in the epoetin-α and placebo groups (25.9 vs 26.7%).

**Table 3:** Treatment-emergent AEs that occurred in the first 24 weeks in ≥5% of patients (safety analysis—treatment phase only)

	Placebo *n* = 45	Epoetin-α *n* = 85
General disorders	17 (37.8%)	31 (36.5%)
Asthenia	5 (11.1%)	12 (14.1%)
Fatigue	3 (6.7%)	8 (9.4%)
Pyrexia	5 (11.1%)	7 (8.2%)
Edema peripheral	5 (11.1%)	3 (3.5%)
Infections and infestations	11 (24.4%)	24 (28.2%)
Nasopharyngitis	2 (4.4%)	6 (7.1%)
Gastrointestinal disorders	8 (17.8%)	24 (28.2%)
Diarrhea	1 (2.2%)	8 (9.4%)
Constipation	0	6 (7.1%)
Metabolism and nutrition disorders	4 (8.9%)	15 (17.6%)
Respiratory, thoracic and mediastinal disorders	4 (8.9%)	13 (15.3%)
Dyspnea	1 (2.2%)	8 (9.4%)
Skin and subcutaneous tissue disorders	4 (8.9%)	12 (14.1%)
Pruritus	0	5 (5.9%)
Musculoskeletal and connective tissue disorders	11 (24.4%)	11 (12.9%)
Back pain	3 (6.7%)	1 (1.2%)
Investigations	7 (15.6%)	10 (11.8%)
Vascular disorders	4 (8.9%)	10 (11.8%)
Blood and lymphatic system disorders	7 (15.6%)	9 (10.6%)
Anemia	5 (11.1%)	5 (5.9%)
Injury, poisoning and procedural complications	5 (11.1%)	8 (9.4%)
Neoplasms benign, malignant and unspecified (including cysts and polyps)	7 (15.6%)	6 (7.1%)
Cardiac disorders	3 (6.7%)	6 (7.1%)

**Table 4:** Treatment-emergent adverse events of toxicity grade 3 or 4 that occurred in the first 24 weeks of the study (safety analysis—treatment phase only)

	Placebo	Epoetin-α
Infections and infestations	2 (4.4%)	5 (5.9%)
Pneumonia	2 (4.4%)	1 (1.2%)
Sinusitis	0	1 (1.2%)
Soft tissue infection	0	1 (1.2%)
Tooth abscess	0	1 (1.2%)
Urosepsis	0	1 (1.2%)
Blood and lymphatic system disorders	2 (4.4%) 1 (2.2%)	1 (1.2%)
Anemia	1 (2.2%)	1 (1.2%)
Thrombocytopenia	1 (2.2%)	1 (1.2%)
Neutropenia	0	1 (1.2%)
Gastrointestinal disorders	1 (2.2%)	3 (3.5%)
Abdominal pain	0	1 (1.2%)
Diarrhea	0	1 (1.2%)
Gastritis	0	1 (1.2%)
Ileitis	0	1 (1.2%)
Esophagitis	0	1 (1.2%)
Vomiting	0	1 (1.2%)
Toothache	1 (2.2%)	0
Vascular disorders	1 (2.2%)	2 (2.4%)
Embolism	0	1 (1.2%)
Systolic hypertension	0	1 (1.2%)
Aortic dissection	1 (1.2%)	0
Musculoskeletal and connective tissue disorders	0	2 (2.4%)
Back pain	0	1 (1.2%)
Pain in extremity	0	1 (1.2%)
Investigations	4 (8.9%)	1 (1.2%)
Blood pressure increased	0	1 (1.2%)
Serum ferritin increased	2 (4.4%)	0
Hemoglobin decreased	1 (2.2%)	0
Lymphocyte count decreased	1 (2.2%)	0
Neutrophil count decreased	1 (2.2%)	0
White blood cell count decreased	1 (2.2%)	0
Injury, poisoning and procedural complications	0	1 (1.2%)
Traumatic brain injury	0	1 (1.2%)
Metabolism and nutrition disorders	0	1 (1.2%)
Diabetes mellitus	0	1 (1.2%)
General disorders and administration site conditions	2 (4.4%)	0
Disease progression	1 (2.2%)	0
Pyrexia	1 (2.2%)	0
Cardiac disorders	1 (2.2%)	0
Arrhythmia	1 (2.2%)	0
Psychiatric disorders	1 (2.2%)	0
Depression	1 (2.2%)	0

**Table 5:** Summary of key safety findings for the first 24 weeks and for the entire study duration (safety analysis set)

	First 24 weeks		Entire study^a^	
	Placebo	Epoetin-α	Placebo	Epoetin-α
Numbers	45	85	24	85
At least 1 treatment-emergent AE	40 (88.9%)	66 (77.6%)	41 (91.1%)	73 (85.9%)
At least 1 treatment-emergent serious AE	8 (17.8%)	22 (25.9%)	10 (22.2%)	35 (41.2%)
At least 1 treatment-emergent Grade 3 or 4 AE	12 (26.7%)	22 (25.9%)	15 (33.3%)	32 (37.6%)
At least 1 treatment-emergent AE leading to study discontinuation	6 (13.3%)	9 (10.6%)	6 (13.3%)	15 (17.6%)
Deaths	1 (2.2%)	4 (4.7%)	1 (2.2%)	7 (8.2%)
At least 1 thrombotic vascular event	0	4 (4.7%)	0	4 (4.7%)
Disease progression (including progression to AML)	4 (8.9%)	11 (12.9%)	4 (8.9%)	14 (16.5%)
Progression to AML	2 (4.4%)	3 (3.5%)	2 (4.4%)	3 (3.5%)

*AML*   acute myeloid leukemia

^a^Includes all data from baseline through Week 52 (i.e., end-of-study visit after end of treatment extension phase [Week 48]) for patients who entered the treatment extension phase. For patients who did not enter the treatment extension phase, an end of study visit that included safety evaluations was performed at Week 28 (i.e., 4 weeks after last dose at Week 24); all data after Week 24 through Week 28 for these patients are included in the entire study period data set

Treatment-emergent severe adverse events (SAE) were reported by 25.9% and 17.8% of patients in the epoetin-α and placebo groups, respectively. Two treatment-emergent SAEs in the epoetin-α group were considered related to study drug by the investigator: thromboembolism (distal deep venous thrombosis, during the first 24 weeks of treatment) and in one patient the anti-erythropoietin antibody testing (after 24 weeks of treatment) was positive during the routine study safety assessment, which led to permanent discontinuation of the study drug. There were no signs of pure red cell aplasia in the patient’s bone marrow and serum erythropoietin remained detectable and reticulocytes were normal at the last available measurement.

No SAE reported in the placebo group was considered by the investigator to be related to the study agent.

AEs that occurred at a frequency of 2% or more in the epoetin-α group or the placebo group were asthenia (14.1 vs 11.1%), fatigue (9.4 vs 2.2%), nasopharyngitis (7.1 vs 4.4%), diarrhea and dyspnea (9.4 vs 2.2%), constipation (7.1% vs 0), and pruritus (5.9% vs 0).

Thrombovascular events were reported in four patients in the epoetin-α group and none of the patients in the placebo group. Three of the events were confirmed as ischemic stroke, embolism, and phlebitis, and the study investigator considered one embolism to be related to the study agent. Two patients had significant risk factors for thrombovascular events including a medical history or ischemic stroke, atrial fibrillation, congestive heart failure, and superficial thrombophlebitis. No further thrombovascular events were reported in the study after Week 24.

Also, during the first 24 weeks, 11 patients in the epoetin-α group (12.9%), and 4 patients in the placebo group (8.9%) experienced disease progression, including 53 [3.5%] AML progressions in the epoetin-α group and 2 [4.4%] in the placebo group. All progressions to AML occurred prior to or at Week 24. After Week 24, three additional patients in the epoetin-α group experienced disease progression (one at Week 44 and two at Week 48) (Table S6).

Five deaths occurred due to TEAE with onset during the first 24 weeks of the study: four in the epoetin-α group (due to AML, sudden death, cachexia, and renal failure) and one in the placebo group (due to AML). None of the deaths was considered by the investigators to be related to study agent.

### Open-label access study: efficacy and safety

Twenty-six patients enrolled in the open-label access study (Table S8), including 10 non-responders in the placebo group after week 24, (7 of whom responded to treatment), and 16 patients responders enrolled after 48 weeks to continue treatment (13 did not relapse) (Table S9).

## Discussion

This is the first placebo-controlled, randomized study assessing epoetin-α (without possible addition of G-CSF) in anemia associated with IPSS low- or intermediate-1-risk MDS. Epoetin-α significantly induced and sustained ER (assessed using IWG-2006 criteria), significantly reduced the RBC transfusion requirements, and prolonged the time-to-first-RBC-transfusion.

ER with epoetin-α was 31.8% strictly applying IWG-2006 criteria, i.e., lower than ER generally reported in phase II trials with epoetin-α at similar dosing schedules in low- and intermediate-1-risk MDS patients [13–17]. In a study by Greenberg et al. also applying strictly IWG 2006 criteria, the ER rate with erythropoietin alone was 34% [13]. On the other hand, in our study, the ER increased to 45.9% after blindly reviewing responses and adjusting response criteria. Application of IWG-2006 criteria was affected by patients who had to transiently discontinue epoetin-α due to a rapid rise in Hb level, and therefore, often had responses shorter than 8 weeks (due to drug discontinuation). Additionally, because only moderately transfused patients could be included, not only RBC transfusion independence, but also an increase in Hb level >1.5 g/dL was required, raising the problem of what baseline Hb level should be chosen in regularly transfused patients. Other difficulties were the differences in values recorded when Hb levels were assessed at local centers compared with centralized assessments. For those reasons, a RRC blindly reviewed all cases, based on modifications of IWG-2006 criteria that took into account drug discontinuations due to “over” response, choosing a pre-transfusion baseline level in transfusion dependent patients, and relying only on a centralized Hb level in case of discrepancies with local Hb level.

After this review, the ER increased from 31.8 to 45.9% in the epoetin-α group and remained at 4.4% in the placebo group. Similar increases in ER after review of IWG 2006 criteria were observed in a recent study of darbepoetin [18]. The results of the open-label access study further support this interpretation, as patients who began treatment with epoetin-α during this phase of the study, had an ER rate of 50% using IWG-2006 criteria.

In this study, ER was not significantly different in the RARS/RCMD-RS vs RA/RCMD MDS subtypes (38.1 vs 30.2%). Our results were also influenced by Nordic Score prognostic factors [19], based on baseline erythropoietin level and RBC transfusion requirement. However, the response rates were lower in our study than those reported by Hellström-Lindberg et al. (e.g., High Nordic Score: 44.7 vs 74%).

The mean duration of response was 27.5 weeks, and the Kaplan–Meier analysis estimated the median duration of response to be 44 weeks, i.e., less than median responses of 20–24 months usually reported in the literature [13–17]. However, this endpoint was difficult to assess in the present study since many patients discontinued treatment while still responding (including patients deemed non responders per strict IWG 2006 criteria).

The safety findings were consistent with the known profile of epoetin-α [4, 13–15, 20–22], or were related to patients’ underlying health conditions.

This study supports the efficacy and safety of epoetin-α in patients with anemia associated with low-risk MDS. Based on these results, the risk-benefit profile of epoetin-α in the treatment of anemia in patients with low-risk MDS is positive. As a result of this study, epoetin-α has been approved by the French ANSM [23, 24].

Finally, IWG-2006 criteria were difficult to apply, as previously experienced in the MDS 005 study of lenalidomide in non-deletion-5q low-risk MDS [23]. IWG criteria for response assessment may have to be modified to be applicable to clinical study design. Amendments to IWG-2006 criteria could include separating RBC transfusion dependent patients into those with low and high RBC-transfusion dependence, defining transfusion dependence over the previous 16 weeks (rather than eight weeks), better defining timing of “baseline Hb level” and taking into account treatment discontinuation due to “over” response, defining a tolerable period (days) of oscillation of Hb with subsequent return to previous levels that does not impact on overall response, and the intervals of measurement of Hb (weekly/biweekly) during treatment.

## Electronic supplementary material

Figure S1

Table S1

Table S2

Table S3

Table S4

Table S5

Table S6

Table S7

Table S8

Table S9
