# Supplementary figures and images for: A phase 3 randomized, placebo-controlled study assessing the efficacy and safety of epoetin-α in anemic patients with low-risk MDS
Source: Leukemia. 2018 Mar 30;32(12):2648–58. doi: 10.1038/s41375-018-0118-9 (PMC6286328; doi:10.1038/s41375-018-0118-9)

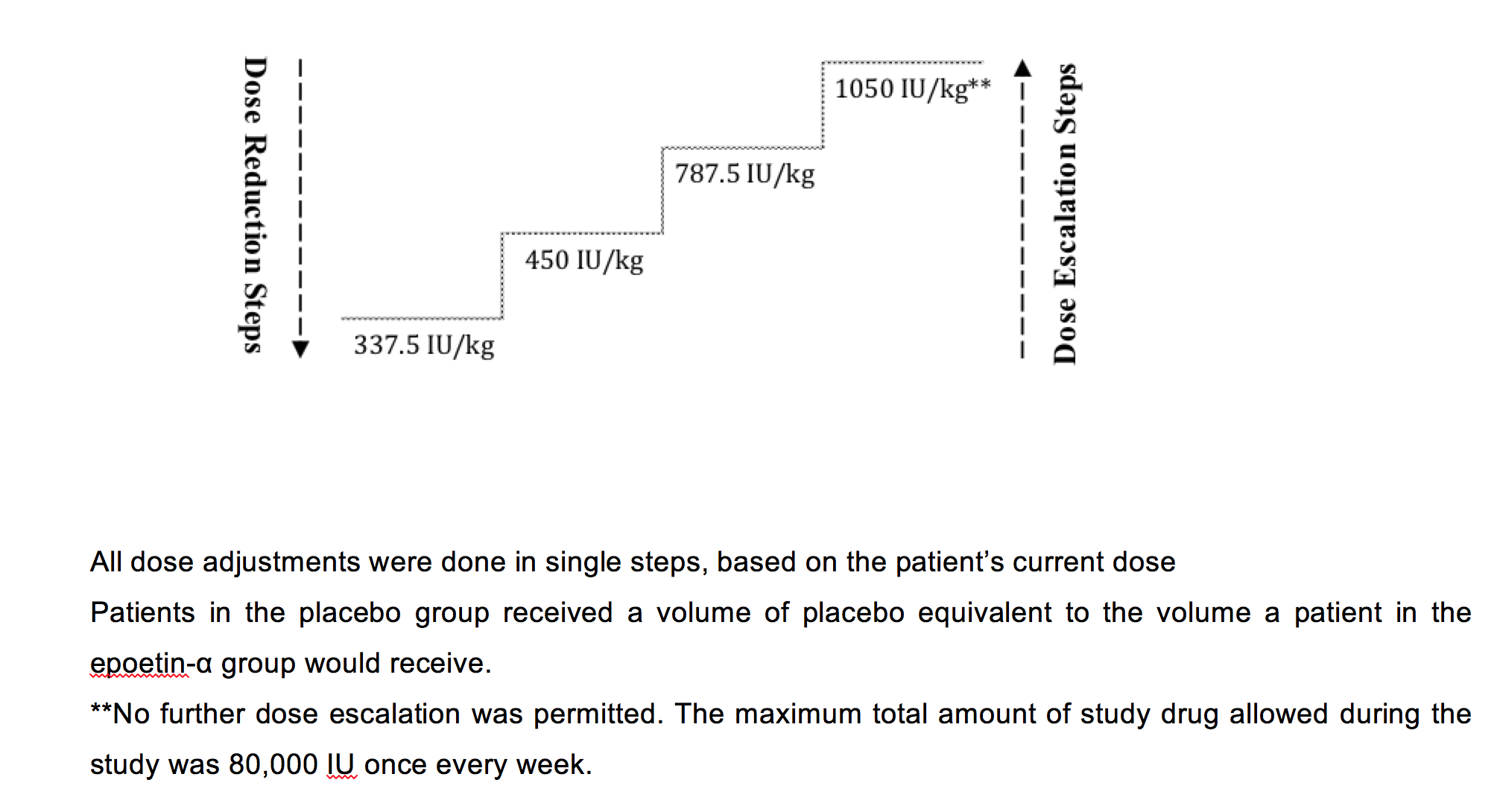

Supplement: Supplementary file 1 — Figure S1 [file 41375_2018_118_MOESM1_ESM.tif]
